# The Natural Occurring Compounds Targeting Endoplasmic Reticulum Stress

**DOI:** 10.1155/2016/7831282

**Published:** 2016-08-03

**Authors:** Hai Liu, Jianqiong Yang, Linfu Li, Weimei Shi, Xiaoliang Yuan, Longhuo Wu

**Affiliations:** ^1^College of Pharmacy, Gannan Medical University, Ganzhou 341000, China; ^2^Department of Clinical Research Center, The First Affiliated Hospital, Gannan Medical University, Ganzhou 341000, China; ^3^Department of Respiratory Medicine, The First Affiliated Hospital, Gannan Medical University, Ganzhou 341000, China

## Abstract

ER stress has been implicated in pathophysiological development of many diseases. Persistent overwhelming stimuli trigger ER stress to initiate apoptosis, autophagy, and cell death. IRE1-JNK and eIF2*α*-CHOP signaling pathways are the two important players of ER stress, which is also modulated by ROS production, calcium disturbance, and inflammatory factors. ER stress has been developed as a novel strategy for diseases management. Recently, a vast of research focuses on the natural occurring compounds targeting ER stress, which results in medical benefits to human diseases. These small reported molecules mainly include polyphenols, alkaloids, and saponins. Many of them have been developed for use in clinical applications. To better understand the pharmacological mechanism of these molecules in ER stress in diseases, efforts have been made to discover and deliver medical merits. In this paper, we will summarize the natural occurring compounds targeting ER stress.

## 1. Introduction

Endoplasmic reticulum (ER) is the organelle functionally responsible for folding proteins, calcium storage, and lipid synthesis. Newly produced proteins are delivered to ER for further modification, which includes glycosylating, folding, and forming disulfide bond events [1]. To ensure the fidelity of protein folding and maturation, ER develops a mechanism for controlling quality, called ER quality control (ERQC). Normally, proteins will be scheduled for inspection by ERQC, or they will be targeted for degradation through ER-associated degradation (ERAD) [2]. When some detrimental stimuli, including glucose deprivation, redox imbalance, and calcium disruption, pose on cells, ER may be accumulated by unfolded or misfolded proteins, which activate ER stress. To self-protect for survival, cells evolve a mechanism called unfolded protein response (UPR) to adapt to the new microenvironment [3]. UPR-associated sensors include inositol-requiring enzyme-1 (IRE1*α*), PKR-like ER kinase (PERK), and activating transcription factor-6 (ATF6), which are the players to restore and maintain the cellular homeostasis [4]. The underlying mechanisms are associated with decreased loading of proteins into ER, attenuation of gene translation, and correction of protein folding [5]. However, once the stress is overwhelming and persisting, cells may initiate the process of apoptosis or cell death [6].

Chinese traditional medicine (TCM) has greatly developed and provided medical benefits to human healthcare. From the history of drug discovery, the natural occurring products have provided us with numerous resources to develop the applicable candidates. Recently, many natural occurring compounds have been found to target ER stress (Table 1), which plays an important role in the pathophysiological development of diseases. Specifically, thapsigargin, produced by* Thapsia garganica*, is considered as an ER stress inducer through noncompetitive inhibition of sarco/endoplasmic reticulum Ca^2+^ ATPase (SERCA). In this paper, we will summarize the natural occurring compounds targeting ER stress.

## 2. The Natural Occurring Compounds Interact with ER Stress

### 2.1. ER Stress-Related Apoptosis

#### 2.1.1. PERK-eIF2*α*-CHOP

Four flavonoid compounds have been separated from the TCM; the root of* Scutellaria baicalensis* Georgi and cytotoxicity assays show that only baicalein at the water-soluble dosage may activate ER stress, leading to inhibition of the viability and colony formation in hepatocellular carcinoma (HCC) cells. Baicalein induces HCC cells apoptosis and autophagy through upregulation of CHOP and JNK expression and downregulation of Bcl-2 family protein expression. The other three flavonoids wogonin, baicalin, and wogonoside cannot reach a satisfactory activity [7]. Berberine is an isoquinoline alkaloid separated from* Rhizoma coptidis* and has been used as a TCM for treating diseases, including dyslipidaemia, obesity, and hyperglycaemia. Berberine functions as an antidiabetic agent to decrease the phosphorylation levels of PERK, eIF2*α*, and IRS-1-ser307 and subsequently reduces ER stress to improve insulin sensitization in Hep G2 cells [8]. It has been reported that celastrol, a natural triterpenoid component from* Tripterygium wilfordii* Hook, may synergize with the BH3 mimetic drug ABT-737 to induce HCC cells apoptosis through upregulation of Noxa expression, which may be activated by eIF2*α*-ATF4 signaling pathway [9].

ERAD and protease-regulated degradation of misfolded nuclear receptor corepressor (N-CoR) promote cells resistance to UPR-induced apoptosis in acute promyelocytic leukemia (APL). Curcumin may inhibit activities of ERAD and protease-regulated degradation and subsequently promote accumulation of phosphorylated misfolded N-CoR, leading to sensitization of APL to UPR-induced apoptosis, which is indicated by upregulation of PERK-eIF2*α* and CHOP [10]. Honokiol is a component isolated from* Magnolia officinalis*. ER stress-induced apoptosis in torsion/detorsion testicular injury may be attenuated by honokiol through downregulation of p-eIF2*α* and CHOP expression [11]. Crocin, a carotenoid separated from* Crocus sativus* L stigma, has been reported to suppress ER stress induced by 1-methyl-4-phenylpyridinium (MPP+) in PC12 cells (Table 1), resulting in inhibition of CHOP-Wnt-mediated apoptosis [12]. Astragaloside IV separated from* Astragalus membranaceus* (Fisch.) Bge has been demonstrated to exhibit protective activity in diabetic nephropathy through inhibition of ER stress [13]. Specifically, astragaloside IV downregulates PERK-ATF4-CHOP signaling to reduce podocyte apoptosis in diabetic rats [14]. Anacardic acid has been reported to be an ER stress inducer in myeloma U266 cells and hepatoma HepG2. Anacardic acid may ATF4-dependently upregulate the expression of eIF2*α*, GRP78, and CHOP expression, resulting in cell apoptosis [15].

Resveratrol may reduce fat accumulation and body weight through regulation of lipid and glucose metabolism. This might be associated with downregulation of AMPK signaling pathway and activation of ER stress, as revealed by increased expression of eIF2*α* phosphorylation and CHOP [16]. The underlying mechanisms of resveratrol inducing ER stress-associated apoptosis might be related to suppression of the hexosamine biosynthetic pathway and interruption of GSK-3*β*-modulated protein glycosylation, which is regulated by Akt and ENTPD5 (ectonucleoside triphosphate diphosphohydrolase 5) in ovarian cancer cells [17]. The notion that GSK-3*β* modulates CHOP activity has been investigated that ginsenoside Rb1 protects neurons from the toxicity of high glucose through inhibition of GSK-3*β*-mediated CHOP expression [18].

#### 2.1.2. GRP78 and IRE1-XBP1

EGCG, an inhibitor of GRP78, possesses both antioxidative and prooxidative activities. It has been demonstrated that EGCG may significantly enhance the therapeutic activity of temozolomide to induce glioblastoma apoptosis through inhibition of GRP78 activity [19]. On the other hand, it has been identified that honokiol possesses much more affinity to GRP78 than EGCG to induce apoptosis in neuroblastoma cells [20]. Cisplatin (CP), an anticancer drug, promotes nephrotoxicity as its side effects, through activation of ER stress. EGCG may significantly reduce the expression of GRP78 and caspase-12, leading to attenuation of ER stress-induced apoptosis in mouse renal tubular epithelial cells [21] (Table 1). Nicotine is the main constituent of tobacco. Nicotine has been demonstrated to decrease apoptosis induced by tunicamycin- (TM-) mediated ER stress in PC12 cells through decreased expression of GRP78, ATF6, and IRE1-XBP1 [22].

Resveratrol is a natural polyphenol separated from red grapes and wine. Resveratrol has* trans* and* cis* isomers. Both isomers have the capability of antioxidation, anti-inflammation, antitumor, and immunomodulation.* Cis*-resveratrol may inhibit GRP78 expression and decrease ROS production in human macrophages [23]. Resveratrol may induce ER stress-induced apoptosis through abrogation of the prosurvival IRE1-XBP1 signaling and activation of the proapoptotic responses [24]. However, resveratrol has been demonstrated to activate ER stress-induced apoptosis through ER expansion and caspase-12 signaling, but not IRE1 or CHOP signaling, in human nasopharyngeal carcinoma cells [25].

#### 2.1.3. Combination

In isolated carotid arteries from spontaneously hypertensive rats, increased ROS production, decreased AMPK phosphorylation, elevated eIF2*α* phosphorylation, and enhanced expression of ATF6, XBP1, and COX-2 are observed. These pathological activities may be reversed by berberine to downregulate endothelium-dependent contractions (EDCs) [26]. Icariin is a flavonoid derived from* Epimedium brevicornum* Maxim. It has been investigated that icariin exhibits cardiovascular protective roles through attenuation of ER stress as indicated by downregulation of GRP94, GRP78, and CHOP expression and ROS generation [27]. In human esophageal squamous cell carcinoma, icariin may activate the expression of ER stress-associated proteins, including p-PERK, p-eIF2*α*, CHOP, GRP78, and ATF4, and downregulate the expression of Bcl-2, leading to sensitization of EC109 and TE1 cells to icariin treatment [28]. Consistently, icariin promotes ER stress-induced apoptosis in human lung adenocarcinoma A549 cells [29] (Table 1).

Curcumin significantly improves the ER stress-induced alternations of cellular morphology and histopathology and inhibits cell apoptosis in liver in streptozotocin-induced diabetic rats [30]. The activities of curcumin in promoting apoptosis associated with ER stress and mitochondrial dysfunction in human gastric carcinoma AGS cells and colon carcinoma HT-29 cells have also been investigated [31]. Consistently, curcumin is also implicated in ER stress-, mitochondrial dysfunction-, and ROS-mediated apoptosis in murine myelomonocytic leukemia WEHI-3 cells [32]. In addition, curcumin induces cell arrest at G2/M phage in human tongue cancer SCC-4 cells through downregulation of cyclin B/CDK1 and CDC25C. Similarly, curcumin induces SCC-4 cells apoptosis in an ER stress- and mitochondrial-dependent manner [33]. Curcumin also exhibits the immunomodulatory activities to promote apoptosis through an excessive ER stress response in human CD4+ and Jurkat T cells [34].

Oxidized low-density lipoprotein (ox-LDL) can produce cytotoxicity on RAW264.7 macrophages and accumulate to trigger ER stress, leading to CHOP-mediated apoptosis. Quercetin may significantly attenuate TM- or ox-LDL-induced ER stress, as indicated that IRE1-XBP1 and ATF6 expressions are decreased [35]. Asymmetric dimethylarginine (ADMA) may induce renal fibrosis through activation of ER stress, including PERK-CHOP and IRE1-JNK signaling pathways, leading to apoptosis in glomerular endothelial cells (GEnCs). Quercetin has been reported to protect against renal damage through attenuation of ER stress [36]. The antioxidant quercetin may synergize with apigenin to ameliorate TM-induced ER stress and mitochondrial dysfunction in adipocytes [37]. Similarly, quercetin also induces caspase-dependent apoptosis in PC-3 cells through activation of ER stress and mitochondrial dysfunction [38]. Quercetin enhances the cytotoxicity of cisplatin in ovarian cancer cells through suppression of STAT3 phosphorylation and activation of ER stress, leading to decreased expression of Bcl-2 and induction of cells apoptosis [39]. Quercetin also sensitizes human ovarian cancer cells to TRAIL and induces cells death through upregulating the expression of CHOP- and JNK-mediated death receptor 5 (DR5), which is regulated by the generation of ROS [40].

Pterostilbene is a natural analogue of resveratrol and exhibits a protective role in human umbilical vein endothelial cells (HUVECs). Pterostilbene downregulates the expression of TNF*α*-induced ER stress proteins, such as GRP78 and p-eIF2*α* [41]. ER stress contributes to vascular degeneration in retina. It has been demonstrated that resveratrol suppresses retinal ischemia/reperfusion and downregulates TM-induced eIF2*α*-CHOP and IRE1*α*-XBP1 signaling pathways [42]. SIRT1 ameliorates insulin resistance through attenuation of mTORC1 expression and ER stress. Resveratrol, a SIRT1 activator, attenuates the TM-induced expression of GRP78, XBP1, and CHOP in a SIRT1-dependent manner [43]. SIRT1 may decrease ER stress-mediated cardiomyocyte apoptosis through involvement of IRE1*α*/JNK, PERK/eIF2*α*, and ATF6/CHOP-mediated signaling pathways [44]. Oplopantriol-A derived from* Oplopanax horridus* preferentially induces cell death in cancer cells. The activity of oplopantriol-A activating ER stress might be associated with ubiquitinated/proteasome signaling pathway. In addition, oplopantriol-A induces cell death that is BH3-only protein-dependent [45]. Similarly, a natural polyyne falcarindiol targets colon cancer cells preferentially through induction of ER stress-related apoptosis, which is also associated with accumulation of ubiquitinated proteins [46].

In human periodontal ligament cells, nicotine may trigger ER stress, as evidence that p-PERK, p-eIF2*α*, CHOP, and GRP78 are significantly upregulated, leading to activation of MMPs, ECM degradation, and cell apoptosis [47]. ER stress has been linked to nicotine-induced placental insufficiency, which may be associated with poor disulfide bone formation [48]. However, in Parkinson's disease, nicotine exhibits neuroprotective activity through suppression of UPR signaling, upregulation of nicotinic acetylcholine receptors (nAChRs) expression [49].

Trichodermin, a fungal metabolite from* N. psidii*, has been demonstrated to induce apoptosis in human chondrosarcoma cells through mitochondrial dysfunction and ER stress, as characterized by increased expression of GRP78, IRE1, GRP94, and p-PERK, and disruption of calcium level [50]. 20(S)-ginsenoside Rg_3_ isolated from* Panax* ginseng promotes TRAIL-induced apoptosis in HCC cell lines, including SK-Hep1, Huh-7, HepG2, and Hep3B, through regulation of CHOP-mediated DR5 at the transcriptional level [51]. Rg_3_ also has been shown to induce ER stress-apoptosis in primary human gallbladder cancer cells, as revealed by upregulation of IRE1/PERK phosphorylation, CHOP, and caspase-12 expression [52].

### 2.2. ROS-Mediated ER Stress

Ischemia/reperfusion injury produces an increased level of ROS, which results in oxidative stress and cells apoptosis. Berberine effectively attenuates ROS-mediated oxidative stress, which activates ER stress as evidenced that GRP78 and CHOP are significantly upregulated in HK-2 cells. In addition, Berberine also decreases mitochondrial-dependent apoptosis through decreasing the ratio of Bax/Bcl-2 [53]. Similarly, berberine exhibits cardioprotective roles against myocardial ischemia/reperfusion jury through activation of JAK2/STAT3 signaling, which negatively regulates ER stress-induced apoptosis [54]. Elatoside C derived from* Aralia elata* shows cardioprotective activity in ischemia/reperfusion induced apoptosis through attenuation of ER stress and increased phosphorylation of STAT3 [55]. Notoginsenoside R1, a component from* P. notoginseng*, attenuates the expression of p-PERK, ATF6, GRP78, IRE1, CHOP, and p-JNK, leading to decreased apoptosis and cardioprotective activity against ischemia/reperfusion damage [56].

Baicalein exhibits both proapoptotic and antiapoptotic activity, depending on the cell types and cell status. In HT22 neuronal cells, baicalein protects against cell apoptosis through reducing the expression of ER stress-associated proteins, including GRP78, CHOP, XBP1, JNK, and ATF6*α*. Meanwhile, baicalein also decreases the production of MMPs and ROS, which cause matrix and cells degeneration. The cytoprotective activity of baicalein may be associated with its ability of quenching free radicals against genotoxicity and cytotoxicity [57]. In contrast, berberine dose-dependently promotes apoptosis through increasing ROS levels in Ca Ski cells, leading to activation of ER stress as characterized by release of Ca^2+^ and upregulation of CHOP [58] (Table 1). Nicotine may trigger ROS production, ER stress, and lipid dysregulation, which are related to age-related macular degeneration (AMD) pathogenesis [59]. Crocetin is a carotenoid and has a similar biological activity with crocin. It has been demonstrated that crocetin may significantly reduce ROS production, maintain the mitochondrial membrane potential, inhibit ER stress, and subsequently decrease apoptosis in retinal ganglion cells [60].

Several polyphenolic compounds, including apigenin (APG), genistein (GST), (−)-epigallocatechin-3-gallate (EGCG), and (−)-epigallocatechin (EGC), have been administrated to human glioblastoma T98G, U87MG, and HNA cells (Table 1). The results show that these natural compounds promote cell apoptosis in T98G and U87MG cells, but not HNA cell, through induction of ROS and elevation of intracellular free Ca^2+^, which activate ER stress [61]. The neuroprotective role of EGCG has been investigated by which EGCG prevents neurological damage in the rat transient focal cerebral ischemia models through inactivation of ER stress in a MEK/ERK-dependent manner [62]. EGCG may enhance glucose-stimulate secretion of insulin, improve islet structure, and attenuate the activity of ER stress, which are possibly associated with the antioxidative activity of EGCG [63].

Similarly, curcumin induces apoptosis in a ROS-dependent manner in cervical cancer cells (C33A, CaSki, HeLa, and ME180) through activation of ER stress. Specifically, curcumin does not elevate ROS level in normal epithelial cells [64]. Hypoxia promotes oxidative stress and ER stress. Peroxiredoxin (Prdx) is one of members in peroxidases family that detoxify lipid peroxides and hydrogen peroxide. Treatment with the chemical agent CoCl_2_ in mouse hippocampal (HT22) cells may produce hypoxia through downregulation of Prdx6 expression, which is regulated by NF-*κ*B signaling pathway, leading to induction of ROS-trigger ER stress-mediated apoptosis. These can be reversed by administration of curcumin to rescue cell [65]. Curcumin may trigger the expression of endogenous antioxidants, which negatively regulate ER stress. Diabetic embryopathy including neural tube defects (NTDs) may be induced by hyperglycemia triggering ROS-mediated ER stress. Curcumin has been investigated to reduce NTD formation through abrogation of ER stress and activation of caspases [66]. ROS mediating ER stress-induced apoptosis has also been involved in breast cancer cells growth inhibition, which is mediated by saxifragifolin D, a compound from* Androsace umbellate* [67].

Patulin can be produced by many species, such as* Penicillium* and* Aspergillus*, and damage intestinal and kidney, which is possibly associated with induction of ER stress and ROS generation. It has been demonstrated that quercetin and crocin may synergize to alleviate ROS-mediated ER stress and lipid peroxidation, leading to maintenance of mitochondrial membrane potential and inhibition of caspases cascade [68]. The combination of quercetin and crocin also attenuates ER stress induced by zearalenone, a secondary metabolite from* Fusarium*, and subsequently inhibits ROS generation, lipid peroxidation, and cells apoptosis [69]. Platycodin D isolated from* Platycodon grandiflorum* has been demonstrated to trigger apoptosis through upregulation of ROS-mediated ASK1 and ER stress in MCF-7 cells [70]. Polyphyllin D separated from* Paris polyphylla* is investigated to induce ER stress-apoptosis in NSCLC cells, as indicated by increased expression of GRP78, CHOP, and caspase-4 [71].

Resveratrol may sensitize human lung adenocarcinoma A549 cells to As_2_O_3_, leading to mitochondrial dysfunction and increased expression of ER stress-associated proteins, including CHOP, GRP78, and caspase-12. The effects of resveratrol and As_2_O_3_-induced ER stress and mitochondrial dysfunction may be blocked by the ROS scavenger N-acetyl-l-cysteine [72]. However, resveratrol has been demonstrated to promote palmitate-induced cell death through reduction of ROS generation and enhancement of CHOP and XBP1 expression in HepG2 cells [73]. Although protein inhibitors benefit antiretroviral management of HIV-1 infection, the adverse side effects induced by ER stress and ROS production are compromising. In myotubes, resveratrol attenuates the deleterious activities, including increased expression of CHOP, elevation of ROS production, altered ER morphology, and reduction of caveolin 3 and flotillin 1 expression at lipid raft [74]. The beneficial effects of resveratrol and omega-3 fatty acid on ROS-mediated ER stress in cardiovascular diseases have been comprehensively reviewed [75].

Withaferin A is natural product from* Withania somnifera* and ROS-dependently induces the expression of GRP78, CHOP, eIF2*α*, and XBP1 in CaSki cells, leading to cell apoptosis [76]. Piperlongumine isolated from pepper plants shows cytotoxic activity to cancer cells and preferentially suppresses HCC cells migration through MAPK/CHOP signaling in ROS-dependent manner [77]. Polydatin may exhibit protective effects against damage from ischemia/reperfusion. Polydatin also induces apoptosis in human nasopharyngeal carcinoma CNE cells through induction of ROS-mediated ER stress, as indicated that the antioxidant N-acetyl cysteine may abrogate the induction of ER stress and cells apoptosis [78]. ERp44 is an ER-resident protein and responsible for oxidative protein folding. Honokiol may directly bind to ERp44 and reduce its activity, leading to activation of ER stress and subsequent apoptosis in HN22 and HSC4 cells [79] (Table 1).

### 2.3. Calcium-Mediated ER Stress

Bisdemethoxycurcumin (BDMC), demethoxycurcumin (DMC), and curcumin are the main constituents for curcuminoids in many* Curcuma* species. BDMC has been demonstrated to induce human lung cancer NCI H460 cells arrest at S phase and subsequent apoptosis through elevation of Ca^2+^ and ROS and activation of ER stress, as indicated that GRP78, IRE1*α*, IRE1*β*, CHOP, ATF6*α*, ATF6*β*, and caspase-4 are significantly upregulated [80]. In retina ganglion cells (N18), baicalein promotes cell apoptosis mediated by ER stress through induction of Ca^2+^ and ROS in the cytoplasm and decreasing mitochondrial membrane potential, leading to the release of cytochrome *c* into cytosol and activation of caspase-3 [81]. Consistently, baicalein induces MDA-MB-231 cell apoptosis through elevation of Ca^2+^, activation of ER stress, downregulation of Bcl-2 expression, upregulation of Bax expression, and reduction of mitochondrial membrane potential [82]. Camphene separated from the essential oil of* Piper cernuum* Vell. leaves has been showed to activate apoptosis in melanoma cells through induction of ER stress, which might be associated with calcium disturbance and mitochondria dysfunction [83].

Honokiol has been demonstrated to induce apoptosis in human chondrosarcoma cells (JJ012 and SW1353) but not primary chondrocytes. Honokiol may disrupt the balance of Ca^2+^ in cytosol and trigger ER stress as indicated by upregulation of GRP78, leading to cell apoptosis through elevation of Bax/Bak expression and attenuation of mitochondria function and Bcl-XL expression [84]. Elevation of intracellular Ca^2+^ promotes ROS activation, leading to cell apoptosis. It has been demonstrated that berberine elevates the concentrations of intracellular Ca^2+^ within 12 h and ROS within 18 h in T98G cells. The disruption of Ca^2+^ in the cytoplasm may be produced by ER stress, as revealed that the ER stress-associated proteins, including GRP78, ATF6, PERK, eIF2*α*, and CHOP, are significantly upregulated by berberine [85]. Gypenosides, triterpenoid saponins separated from* Gynostemma pentaphyllum* Makino, have been demonstrated to activate apoptosis through calcium-modulated ER stress and mitochondrial dysfunction in human hepatoma cells [86].

In cancer cells, such as MDA-MB 435S, MCF-7, DLD-1, and RKO cells, IP3R-mediated Ca^2+^ is released from ER and mitochondrial overloading of Ca^2+^ triggered by celastrol promote paraptosis (Table 1). This homeostatic imbalance of Ca^2+^ induces misfolded proteins accumulation within ER, which results in impaired activities of chaperones and protein processing [87]. Curcumin may synergize with carnosic acid to induce apoptosis in acute myeloid leukemia (AML) cells in a cancer-selective cytotoxicity manner through disruption of Ca^2+^ homeostasis. Results also indicate that intracellular Ca^2+^ mobilization is required but not sufficient for inducing AML apoptosis. However, ER stress and mitochondrial damage are not involved in this mechanical regulation [88]. Calreticulin, a Ca^2+^-binding protein, acts as a multifunctional chaperone located in ER and plays a critical role in cancer cell growth and peritoneal dissemination. Honokiol may significantly downregulate the expression of calreticulin and PPAR*γ* through activation of ER stress [89].

### 2.4. Inflammation-Mediated ER Stress

ER stress may promote inflammatory responses. Curcumin has been reported to exhibit anti-inflammatory activities against the invasion of bacteria in intestinal epithelial cells (Caco-2 cells and T84 cells) through downregulation of ER stress, as evidenced by decreased expression of GRP78 and IRE1*α*-XBP1 signaling [90]. Sterol ergosta-7,22-dien-3-ol derived from the Echinoderm* Marthasterias glacialis* has been demonstrated to exhibit anti-inflammatory activities, as indicated by upregulation of COX-2, iNOS, IL-6, and NF-*κ*B. It has been demonstrated that CHOP-modulated ER stress upregulates the inflammatory responses, which may be attenuated by sterol ergosta-7,22-dien-3-ol [91].

Investigation shows that berberine promotes Caco-2 cells to survive from ER stress induced by IFN-*γ*/TNF-*α* and TM through increased expression of GRP78 and XBP1 and decreased expression of JNK phosphorylation and caspase-3 and caspase-12 [92]. Berberine also has been investigated for the therapeutic effects on HIV protease inhibitor-induced inflammatory activities through attenuation of UPR and ERK signaling [93]. In experimental autoimmune encephalomyelitis (EAE), crocin may improve neurobehavioral outcomes through suppressing ER stress and inflammatory gene expression in spinal cords, which are mediated by syncytin-1 and NO [94].

ER stress-associated TXNIP/NLRP3 inflammasome leads to inflammation and cell death in the endothelial dysfunction. EGCG, quercetin, and luteolin have been reported to activate AMPK signaling, resulting in inhibition of ER stress and TXNIP/NLRP3 inflammasome [95]. Glutamate promotes neurotoxicity and damage in the hippocampus through induction of ER stress and ROS generation and activation of TXNIP/NLRP3 inflammasome. Curcumin has been demonstrated to attenuate glutamate neurotoxicity through activation of AMPK signaling, which decreases ER stress and inhibits TXNIP/NLRP3 inflammasome through phosphorylation and accelerated degradation [96]. Ilexgenin A may suppress ROS-mediated TXNIP/NLRP3 inflammasome induction and thus inactivate ER stress through activation of AMPK, leading to protection for endothelial functions in cardiovascular diseases [97] (Table 1). Similarly, mangiferin also attenuates ER stress-associated TXNIP/NLRP3 inflammasome in endothelial cells through regulation of ROS production and AMPK phosphorylation [98]. In addition, astragaloside IV may synergize cycloastragenol to suppress ROS-mediated ER stress and inactivate TXNIP/NLRP3 inflammasome through regulating AMPK signaling pathway, leading to endothelial homeostasis [99].

ROS and NF-*κ*B signaling inhibition are involved in anti-inflammatory effects of honokiol. ER stress activation as revealed by upregulation of eIF2*α* and CHOP expression and subsequent cell apoptosis are activated by honokiol in attenuating the severity of acute pancreatitis [100]. In chronic restraint stress, honokiol exhibits neuroprotective activity and inactivates ER stress through downregulation of GRP78, CHOP, and proinflammatory factors expression [101].

### 2.5. ER Stress-Related Autophagy

Autophagy and apoptosis may be the two results of ER stress. Investigation shows that eukaryotic elongation factor-2 kinase (EEF2K) may control cell destination to autophagy or apoptosis, depending on the residues for phosphorylation. Curcumin may promote cells to sensitize apoptosis through inhibition of EEF2K activity [102]. Homeostatic imbalance of calcium storage and store operated calcium entry (SOCE) may trigger ER stress, subsequently activate AMPK and inhibit the Akt/mTOR signaling. Resveratrol has been reported to trigger autophagic cell death in PC3 and DU145 cells through regulation of SOCE signaling and induction of ER stress induced by depletion of calcium pool [103]. Nicotine may trigger an influx of extracellular Ca^2+^, elevate mitochondrial ROS production, and upregulate PI3K-dependent expression of COX-2 and PGE2, leading to activation of autophagy in colon cancer cells [104].

ER stress is implicated in dental fluorosis. It has been demonstrated that fluoride may activate autophagy and protect cells from fluoride-induced toxicity through upregulation of SIRT1. Fluoride inducing upregulation of autophagy associated genes (*Atg*) expression may be activated by resveratrol in ameloblasts [105]. Bortezomib and MG132 are proteasome inhibitors and induce cancer cells death through activation of ER stress. However, it has been found that EGCG may decrease the activity of bortezomib, but not MG132, to ameliorate ER stress and lead to autophagy in PC3 cells. Inhibition of autophagy may restore cancer cells sensitization to ER stress induced by bortezomib and EGCG [106].

Alisol B separated from* Alisma orientale* is investigated as a novel inducer of autophagy. Studies show that alisol B may trigger calcium mobilization and subsequently leads to autophagy through upregulation of CaMKK-AMPK, a target of rapamycin signaling pathway. In addition, alisol B also triggers ER stress-induced apoptosis [107]. Zerumbone is a natural monocyclic sesquiterpene from* Zingiber zerumbet* Smith. It has been investigated that zerumbone may induce apoptosis and autophagy in PC-3 and DU-145 cells through ER stress induction and mitochondrial dysfunction [108].

Vacuolation-mediated cell death may differ from apoptotic and autophagic-death. It has been demonstrated that curcumin promotes vacuolation-mediated cell death through the possible mechanisms of inducing ROS and upregulating CHOP and GRP78 expression [109].

Autophagy may either induce cell death or promote cell survival. Autophagy is a process of cellular degradation, being associated with the turnover of misfolded proteins and recycling nutrients to maintain energy levels for survival. IRE1 deficiency or JNK inhibitors may induce inhibition of autophagy triggered by ER stress. In contrast, PERK deficiency and ATF6 knockdown shows autophagy induction in response to ER stress. These indicate autophagy plays an important role in cell survival after ER stress [110]. Similarly, glycyrrhetinic acid (GA), a main constituent of* Glycyrrhiza uralensis* Fisch., also promotes cytoprotective autophagy in non-small lung cancer cells through modulating the activity of IRE1-JNK/c-jun signaling pathway [111]. Vitexin acts as an antihyperthermic compound to exhibit a cytoprotective role in A549 cells through upregulation of MAPKs, Hsp90, and antioxidant enzyme, resulting in autophagic survival via ER stress-mediated signaling pathways [112]. Autophagic dysfunction induced by natural compounds in prostate cancer might represent a new therapeutic strategy, which has been reviewed [113].

## 3. Miscellaneous

Neurite outgrowth is energy-required and important for nervous system development. In the early stage of polarization, berberine transports across the blood-brain barrier to enhance the ratio of AMP/ATP and activate AMPK signaling to inhibit neurite outgrowth. ER stress and mitochondrial dysfunction are also involved to contribute to the lowered energy status. Interestingly, berberine activates PI3K-Akt signaling to inactivate GSK-3*β*, leading to decreased phosphorylation of tau and decreased stability of microtubule [119]. In C6 rat glioma cells, berberine induces G2/M cell cycle arrest through inhibiting Cdc25c, CDK1, and cyclin B. Meanwhile, berberine elevates the production of ROS and Ca2^+^, induces ER stress, resulting in enhancement of Bax/Bcl-2 ratio, disruption of mitochondrial membrane potential, and cell apoptosis [114] (Table 1).

Celastrol has been demonstrated to decrease the proliferation and increase cell apoptosis in oral squamous cell carcinoma (OSCC) cells through ER stress activation and increased polyubiquitinated proteins accumulation [115]. To screen the targets proteins of celastrol in human lymphoblastoid cells, a large-scale quantitative proteomics has been investigated such that 158 of almost 1800 proteins with 1.5-fold elevation induced by celastrol are identified [120]. The possibly specific targets for celastrol to induce apoptosis mediated by ER stress have been investigated. The results show that proteasome catalytic subunit *β*1 may be the direct target, and mitochondrial import receptor Tom22 (TOM22) and endoplasmic reticulum protein 29 (ERP29) may be the indirect targets. Interestingly, GSK-3*β* is involved in the signaling pathways of celastrol, as evidenced that LiCl may decrease cell apoptosis induced by celastrol [121]. The promotion of proteasome function in inducing cell death by celastrol has been consistently supported. ER stress and Hsp90 inhibition are also synergically involved in induction of paraptosis, autophagy, and apoptosis mediated by celastrol [122].

Activation of AMPK triggers the gene expression of transcriptional corepressor small heterodimer partner-interacting leucine zipper protein (SMILE). The domain in the C terminus of SMILE responsible for repressive activity is corresponding to bZIP domain through which SMILE interacts with cAMP responsive element-binding protein H (CREBH), leading to inhibition of its transcriptional activity. Interestingly, SMILE competes with the coactivator peroxisome proliferative-activated receptor *α* (PGC-1*α*) to bind to CREBH, instead of ATF6. Curcumin has been demonstrated to promote SMILE expression and inhibit CREBH transactivation through activation of AMPK signaling [123]. Curcumin may regulate the activity of transcription factors, including NRF2, CREBH, CREB, SREBP1/2, and PPAR*γ*, which are involved in oxidative stress and lipid homeostasis. These have been reviewed comprehensively [124].

Synoviolin is an ER-anchoring E3 ubiquitin ligase to suppress ER stress-induced apoptosis. The nuclear factor erythroid 2-related factor 1 (NFE2L1) may bind to the distal region of synoviolin promoter and activate its expression. Icariin may decrease apoptosis induced by ER stress through synoviolin/NFE2L1 signaling in PC12 cells [117] (Table 1). Investigation on* Trembler-J* (*Tr-J*) mouse model shows that the genes expression responding to heat-shock proteins, hypoxia, and inflammatory stimuli is significantly upregulated, leading to activation of ER stress and UPR. Curcumin may attenuate the severity of the* Tr-J* neuropathy through upregulation of Hsp70 expression [125]. Synergized with perifosine, an inhibitor of Akt and PI3K, curcumin elevates the level of ROS and attenuates Bcl-2 and cyclin D1 expression in colorectal cancer cells, leading to activation of ER stress and JNK signaling pathway [126].

In an alcohol-induced hepatic fibrosis animal model, curcumin has been demonstrated to play a hepatoprotective role in prevention of fibrosis development. It also inhibits cells proliferation and promotes cell death through ER stress activation and TGF-*β*/Smad signaling attenuation [127]. Nicotine has been reported to lose body weight through decreasing feeding and increasing thermogenesis of brown adipose tissue. This activity might be associated with activation of ER stress [128]. Astaxanthin has been investigated as a beneficial agent for ameliorating insulin resistance with the possible mechanisms that astaxanthin may attenuate ER stress, ROS production, and lipid accumulation, accompanied by decreased expression of JNK1 and NF-*κ*B signaling [129].

Quercetin has been identified as an agonist that binds to IRE1*α* in an unanticipated binding pocket at the dimer interface of IRE1's kinase extension nuclease domain [130]. Quercetin activating IRE1 RNase activity triggers the activation of AMPK signaling, which may be induced by nitric oxide (NO) in pancreatic *β*-cells [118]. The growth arrest and DNA damaged-inducible gene (GADD) 34-mediated phosphorylated eIF2*α* and ATF4 are important for *γ*-secretase activity to produce amyloid *β* (A*β*), which is critical for Alzheimer's disease pathogenesis. It has been demonstrated that quercetin may downregulate *γ*-secretase and A*β* through induction of GADD34, leading to memory improvement in aged mice [131]. Hsp70/Hsp72 has been reported to be a component of UPRsome through binding to the cytosolic domain of IRE1*α*, leading to enhancement of XBP1-mediated protective activity. Quercetin may bind to the region of Hsp70 gene promoter and inhibit its expression. Silence of Hsp70 downregulates the expression of GRP78, but not of CHOP, and induces apoptosis in human leukemia U937 cells [132] (Table 1). These results support the investigation that quercetin induces T98G cells and MOGGCCM cells to be vulnerable to apoptosis through silencing Hsp27 and Hsp72 [133].

Glycyrrhetinic acid derived from liquorice may induce ER stress-associated proteins expression, including GRP78, PERK, and ERP72. This may attenuate protein synthesis, leading to cell cycle arrest in G1 phase in NSCLC cells [116]. Similarly, piperine, an alkaloid from black pepper, is shown to cause HT-29 cells arrest at G1 phase through regulation of cyclin D1/D3 and CDK4/CDK6 and induce ER stress-associated apoptosis through increased expression of CHOP, GRP78, JNK, and IRE1*α* [134].

## 4. Conclusion

The natural occurring compounds, including polyphenols, alkaloids, and saponins, target ER stress in the pathophysiological development of diseases through multiple signaling pathways (Table 1). However, some of them seem to have controversial biological functions in ER stress. This might be associated with the different cell lines and the cells status. IRE1-JNK and eIF2*α*-CHOP signaling pathways are the two important players of ER stress, which are also modulated by ROS production, calcium disturbance, and inflammatory factors. Apoptosis and autophagy may be the two results of ER stress, which have now been developed as a novel strategy for diseases management. Chinese traditional medicine has provided us with a vast of natural compounds most of which are still in screening process. Greater efforts should be made for delivering medical merits.

## Figures and Tables

**Table 1:** The natural compounds biologically targeting different cell lines in ER stress.

Natural products	Cell types [Ref]	Biological functions
Anacardic acid	U266, HepG2 [15]	GRP78↑, eIF2*α* ↑, CHOP↑

Astragaloside IV	Podocyte [14]	PERK-ATF4-CHOP↓

Baicalein	HCC [7]	JNK↑, CHOP↑, Bcl-2 family↓
	Ca Ski [58], N18 [81], MDA-MB-231 [82]	ROS↑, CHOP↑, Ca^2+^↑, caspase-3↑, Bax↑
	HT22 [57]	JNK↓, CHOP↓, GRP78↓, XBP1↓, ATF6*α* ↓, ROS↓, MMPs↓

Berberine	Hep G2 [8]	p-PERK↓, p-eIF2*α* ↓
	Carotid arteries [26], T98G [85]	eIF2*α* ↑, ATF6↑, ROS↑, XBP1↑, COX-2↑, AMPK↓, Ca^2+^↑
	C6 rat glioma cells [114]	ROS↑, Ca^2+^↑, Bax/Bcl-2↑

BDMC	NCI H460 [80]	Ca^2+^↑, ROS↑, ER stress↑

Celastrol	HCC [9]	eIF2*α*-ATF4↑
	MDA-MB 435S, MCF-7, DLD-1, and RKO [87], OSCC [115]	Ca^2+^↑, ER stress↑

Crocin	PC12 [12]	CHOP-Wnt↓

Curcumin	APL [10], HT-29 [31], AGS [31], WEHI-3 [32], SCC-4 [33]	PERK-eIF2*α* ↑, CHOP↑, ER stress↑

EGCG	Glioblastoma [19]	GRP78↓
	T98G [61], U87MG [61]	ROS↑, ER stress↑
	PC3 [106]	Autophagy↑, ER stress↑

Elatoside C	H9c2 cardiomyocytes [55]	ER stress↓, p-STAT3↑

Ginsenoside Rb1	Neurons [18]	CHOP↓

Glycyrrhetinic acid	Non-small lung cancer cells [111]	IRE1*α* ↑, JNK/c-jun↑
	NSCLC cells [116]	GRP78↑, PERK↑, ERP72↑

Honokiol	In vivo [11]	p-eIF2*α* ↓, CHOP↓
	HN22 [79], HSC4 [79], JJ012 [84], SW1353 [84]	ER stress↑, Ca^2+^↑, GRP78↑

Icariin	EC109 [28], TE1 [28], A549 [29]	GRP78↑, p-PERK↑, p-eIF2*α* ↑, CHOP↑, ATF4↑
	PC12 [117]	ER stress↓

Mangiferin	Endothelial cells [98]	TXNIP/NLRP3 inflammasome↓, ER stress↓

Nicotine	PC12 [22]	GRP78↓, ATF6↓, IRE1-XBP1↓
	Colon cancer cells [104]	ROS↑, COX-2↑, PGE2↑
	Human periodontal ligament cells [47]	p-PERK↑, p-eIF2*α* ↑, CHOP↑, GRP78↑

Notoginsenoside R1	H9c2 cardiomyocytes [56]	GRP78↓, p-PERK↓, ATF6↓, IRE1↓, CHOP↓, p-JNK↓

Piperine	HT-29 [117]	CHOP↑, GRP78↑, JNK↑, IRE1*α* ↑

Piperlongumine	HCC [77]	MAPK↑, CHOP↑, ROS↑

Platycodin D	MCF-7 [70]	ROS↑, ASK1↑, ER stress↑

Polyphyllin D	NSCLC [71]	CHOP↑, GRP78↑, caspase-4↑

Pterostilbene	HUVECs [41]	GRP78↓, p-eIF2*α* ↓

Quercetin	RAW264.7 [62], GEnCs [36]	IRE1-XBP1↓, ATF6↓, PERK-CHOP↓, IRE1-JNK↓
	PC-3 [38], ovarian cancer cells [39]	ER stress↑, STAT3↓, JNK↑, CHOP↑, DR5↑, ROS↑
	Pancreatic *β*-cells [118]	AMPK↑, IRE1 RNase↑

Resveratrol	Human skeletal muscle cells [16], human macrophages [23]	eIF2*α* ↑, CHOP↑, AMPK↓, caspase-12↑, GRP78↓, IRE1-XBP1↓
	Cardiomyocyte [44]	IRE1*α*/JNK↑, PERK/eIF2*α* ↑, ATF6/CHOP↑
	A549 [72]	GRP78↑, CHOP↑, caspase-12↑
	DU145 and PC3 [103]	Autophagy↑

Rg_3_	HCC [51], human gallbladder cancer cells [52]	CHOP↑, DR5↑, CHOP↑, p-IRE1↑, p-PERK↑, caspase-12↑

Trichodermin	Chondrosarcoma cells [50]	IRE1↑, GRP78↑, GRP94↑, p-PERK↑

Vitexin	A549 [112]	MAPKs↑, Hsp90↑, antioxidant enzyme↑

Withaferin A	Caki [76]	GRP78↑, eIF2*α* ↑, CHOP↑, XBP1↑

Zerumbone	PC-3 and DU-145 [108]	Apoptosis↑, autophagy↑
